# Portomesenteric Vein Thrombosis After Laparoscopic Sleeve Gastrectomy: Incidence, Analysis and Follow-Up in 1236 Consecutive Cases

**DOI:** 10.1007/s11695-016-2183-3

**Published:** 2016-04-14

**Authors:** Rodrigo Villagrán, Gabriela Smith, Walter Rodriguez, Carlos Flores, Mario Cariaga, Sofía Araya, Marisol Yañez, Paulina Fuentes, Jeannette Linares, Antonio Zapata

**Affiliations:** 1 Bariatric Surgery Department, Bupa Antofagasta Clinic, Matta 1868, 2nd floor, Antofagasta, Chile; 2 Department of Radiology, Bupa Antofagasta Clinic, Antofagasta, Chile; 3 Endocrinology Department, Bupa Antofagasta Clinic, Antofagasta, Chile; 4 Faculty of Medicine and Dentistry, Antofagasta University, Antofagasta, Chile

**Keywords:** Portal thrombosis, Splenic thrombosis, Bariatric complications, Sleeve gastrectomy, Thrombophilia, Bariatric thrombosis

## Abstract

**Background:**

Portomesenteric vein thrombosis (PMVT) is a rare but severe complication after laparoscopic bariatric surgery, with potentially serious consequences. We aimed to describe the incidence, clinical features, management, outcome, and midterm follow-up in patients with PMVT after laparoscopic sleeve gastrectomy (LSG).

**Methods:**

This retrospective and descriptive study included patients who underwent LSG between November 2009 and July 2015 and developed PMVT. The following data were analyzed: age, gender, body mass index (BMI), thrombosis risk factors, surgical technique, thromboembolic prophylaxis, primary surgery outcomes, clinical features, treatment, thrombophilia testing results, and follow-up findings, including imaging and endoscopic findings.

**Results:**

A total of 1236 patients underwent LSG, and 5 (0.4 %) developed PMVT. The mean age was 34.4 years, and 3 patients were women. The mean BMI was 38.5 kg/m^2^. Two patients had received hormonal contraceptive treatment. Four patients had a history of smoking. All of the patients received anticoagulant treatment, and none required surgery. The mean hospitalization duration was 7.6 days. Two patients showed complete recanalization. One patient showed portal cavernomatosis on delayed images. Two patients had a positive thrombophilia test. No portal hypertension endoscopic findings were observed.

**Conclusions:**

PMVT is a rare complication, for which smoking was identified as a predominant risk factor. Early diagnosis and prompt anticoagulant therapy could lead to a dramatic decrease in the incidence of intestinal infarction, mortality, and extrahepatic portal hypertension in the near future. However, careful follow-up is necessary to evaluate the impact of PMVT on long-term patient outcomes.

## Introduction

Portomesenteric vein thrombosis (PMVT) is an uncommon but potentially catastrophic clinical complication that may lead to intestinal ischemia and infarction [1, 2]. Potential etiologies for thrombosis include predisposing factors such as surgery or intra-abdominal inflammation, blunt abdominal trauma, hypercoagulable states, portal hypertension, oral contraceptive use, and bowel obstruction, among others [1–3].

Patients undergoing bariatric surgery are at an increased risk for venous thromboembolism (VTE) due to the underlying inflammatory and hypercoagulable states and because metabolic syndrome may predispose patients to VTE [4, 5]. PMVT has been observed after surgical procedures, including liver transplantation, splenectomy, and bariatric surgery [6–8]. Although significant progress has been made in bariatric surgery and it is becoming more commonly performed due its benefits, it is not exempt from complications that can lead to high morbidity and mortality [9, 10].

During a literature search, we identified a limited number of case reports and series describing PMVT following laparoscopic bariatric surgery as an uncommon complication [11–31].

## Material and Methods

This is a retrospective study of a prospectively collected database of patients who underwent LSG for morbid obesity and developed PMVT as a procedure-related complication. Surgery was performed at the Obesity Surgery Department, Bupa Antofagasta Clinic, Antofagasta, Chile, between November 2009 and July 2015.

The following variables were studied: age, gender, body mass index (BMI), thrombosis risk factors, surgical technique, thromboembolism prophylaxis, primary surgery details, PMVT clinical features, diagnosis methods, treatment, and follow-up findings, including thrombophilia testing results, imaging, and endoscopic findings.

### Surgical Technique

We performed 5 incisions; pneumoperitoneum was created using the closed access technique and a Veress needle, with a maximum preset pressure of 15 mmHg. A liver retractor was placed through a 5-mm incision in the epigastrium. The gastrosplenic ligament as well as gastrocolic omentum were divided using a vessel sealer and divider (LigaSure Atlas®; Covidien Ltd., Norwalk, CT, USA). To calibrate the gastrectomy, a 34 Fr bougie was used. Gastric resection was performed using a gastrointestinal flexible endo-stapler (EndoGIA Roticulator®; Covidien Ltd., Norwalk, CT, USA). A bioabsorbable staple line reinforcement was routinely used (Seamguard® W.L. Gore & Associates, Flagstaff, AZ, USA), and additional clips were placed to control persistent bleeding. The resected stomach was removed through the supraumbilical port site, and we did not routinely use a retrieval bag.

### Multimodal Thromboembolism Prophylaxis Protocol

Intermittent sequential pneumatic compression (ISPC) was used on inferior limbs, starting with the induction of anesthesia and continuing up to 24 h postoperatively. Graduated compression stockings (GCSs) were used beginning after hospital admission and continuing during the entire hospitalization, and they were recommended for 7 days after discharge. Treatment with the low-molecular-weight heparin (LMWH) enoxaparin (Clexane®; Sanofi-Aventis, Paris, France) at 40 mg per day was initiated intraoperatively and continued for 7 days. In January 2015, we modified our protocol in an attempt to reduce complication rates; thereafter, LMWH therapy was extended for up to 14 days. Patients start walking within 4–6 h of surgery with assistance.

In all of the patients who developed PMVT, we conducted follow-up computed tomography (CT). Additionally, upper gastrointestinal endoscopy (UGE) was performed to identify signs of portal hypertension.

The thrombophilia test was performed remotely at initial presentation and at least 2–3 weeks after the cessation of anticoagulation therapy [13].

The institutional review board in our hospital approved this study.

## Results

Among the 1578 laparoscopic bariatric surgeries performed, 1236 (78 %) patients underwent LSG between November 2005 and July 2015 at the Bupa Antofagasta Clinic; five (0.4 %) of these patients developed PMVT. The mean age was 34.4 years (range 27–43 years). Three patients were women. The mean BMI was 38.5 kg/m^2^ (range 36.1–40.9 kg/m^2^). Four patients had a history of smoking (smoking cessation was instructed at least 6 weeks preoperatively), two used hormonal contraceptives, and none had a history of previous thrombosis or family thrombophilia records. No patient had a history of liver cirrhosis. The patient characteristics are summarized in Table 1.

**Table 1:** Patient’s characteristics and first procedure details

Case	Age	Sex	BMI	Comorbidities	Hormonal contraceptive	Smoking history	Thrombosis history or thrombophilia family records
1	43	F	37.4	HTN, IR, FL	Oral	(−)	(−)
2	37	F	36.1	IFG, IR, FL, DLP	Subdermal implant	(+)	(−)
3	27	F	39.0	IFG, IR, FL, DLP	No	(+)	(−)
4	29	M	39.1	HU, IR, DLP	n/a	(+)	(−)
5	36	M	40.9	IFG, IR, FL, DLP, OSA	n/a	(+)	(−)

*F* female, *M* male, *HTN* hypertension, *IR* insulin resistance, *FL* fatty liver, *IFG* impaired fasting glucose, *DLP* dyslipidemia, *HU* hyperuricemia, *OSA* obstructive sleep apnea, *n/a* not applicable

During the LSG procedure, no complications were observed, and no conversion to open surgery was needed. The mean operation room time was 93 min (range 60–135 min). A liver retractor was not necessary in one patient due to the patient’s liver anatomy. All of the patients received complete thromboembolic prophylaxis. One of the patients who developed PMVT received 14 days of treatment due to improvements in the thromboembolic prophylaxis protocol. All of the patients were routinely encouraged to walk with assistance early after surgery; intravenous fluid therapy was also administered. No complications were observed during hospitalization. All of the patients were discharged 48–72 h after surgery. The first procedure and duration details are summarized in Table 2.

**Table 2:** First procedure and stay details

Case	LMWH enoxaparin doses	LMWH 1st doses	LMWH length (days)	OR time (min)	Liver retractor	LOS (hours)
1	40 mg/day	Anesthesia induction	7	90	Nathanson	72
2	40 mg/day	Anesthesia induction	7	60	Nathanson	72
3	40 mg/day	Anesthesia induction	7	105	Nathanson	48
4	40 mg/day	Intraoperatory	7	75	Nathanson	48
5	40 mg/day	Intraoperatory	14	135	No	48

*LMWH* low-molecular-weight heparin, *OR* operation room, *LOS* length of stay

The patients were readmitted at a median of 20 days (range 11–50 days) postoperatively. The main symptom was abdominal pain in all of our patients, starting at 3.4 days (range 2–5 days) before emergency room consultation. Four of five patients showed elevated inflammatory parameters such as the white blood cell count (WBC) and C-reactive protein (CRP) levels at diagnosis. All of the cases were diagnosed by abdominal and pelvic CT enhanced with oral and intravenous contrast according to the emergency unit flowchart for bariatric readmission patients. The patients were admitted to the intensive care unit and immediately underwent intravenous unfractionated heparin therapy, followed by strict medical and CT or ultrasound follow-up, considering the high risk of thrombosis progression to mesenteric veins and small bowel infarction. The therapy was successful, and surgical exploration was not needed. The patients showed satisfactory recovery and remission of the symptoms after 62.4 h (range 48–96 h), and oral intake was progressively resumed. All of the patients were discharged without symptoms after a mean hospitalization period of 7.6 days (range 6–10 days). Anticoagulation therapy with acenocoumarol or warfarin was started after 3.5 days (range 2–5 days) and continued for 4–6 months. One patient continued long-term therapy. The clinical features and treatment details of PMVT are summarized in Table 3.

**Table 3:** Portal thrombosis symptoms, readmission, and treatment. Follow-up with upper gastroscopy and thrombophilia screening

Patient	1	2	3	4	5
Symptoms: F: fever, N: nauseas Pain location	F(−)	F(−)	F(+)	F(−)	F(−)
	N(−)	N(−)	N(−)	N(−)	N(+)
	RL, RH	RH	LL	E	E
Start symptoms before readmission	5	2	3	4	3
POD Readmission	15	50	11	12	16
WBC count (×10^3^/mm3)	6.9	5.2	15.7	12.1	14.7
C-reactive protein (mg/L)	81.9	1	215.3	199.6	61.7
Hematocrit (%)	43.8	39.3	41.9	45.7	46.3
Hemoglobin (g/dL)	15.2	13.3	14.1	16.0	15.9
Coagulation tests PT/	12.2/	12.4/	13.5/	13.6/	12.3/
APTT (seg)	27.2	53.6	28.5	33.6	28.2
AST/ALT levels (U/L)	61/	9/	13/	23/	29/
	120	12	19	33	86
Bilirubin levels	0.6/	0.7/	0.2/	1/	0.3/
Unconjugated/conjugated (mg/dL)	0.6	0.3	0.2	0.7	0.2
Hours at remission of symptoms	96	48	48	72	48
Anticoagulant Therapy	Ac	Ac	Wf	Ac	Wf
Length of stay (days)	7	8	10	6	7
Anticoagulant therapy length (months)	Indefinitely	4	6	6	6
Esophageal varices at UGE	(−)	(−)	(−)	(−)	(−)
(POD, months)	22	12	25	17	2
Thrombophilia screening	ProtC def	n/a	n/a	ProtC def	Neg

*F* fever, *N* nauseas, *RL* right lumbar, *RH* right hypochondrial, *LL* left lumbar, *E* epigastric, *POD* postoperative day, *AST* aspartate transaminase, *ALT* alanine transaminase, *PT* prothrombin time, *APTT* activated partial thromboplastin time, *Ac* acenocoumarol, *Wf* warfarin, *def* deficit, *Neg* negative, *UGE* upper gastrointestinal endoscopy, *n/a* not available

**Fig. 1:**
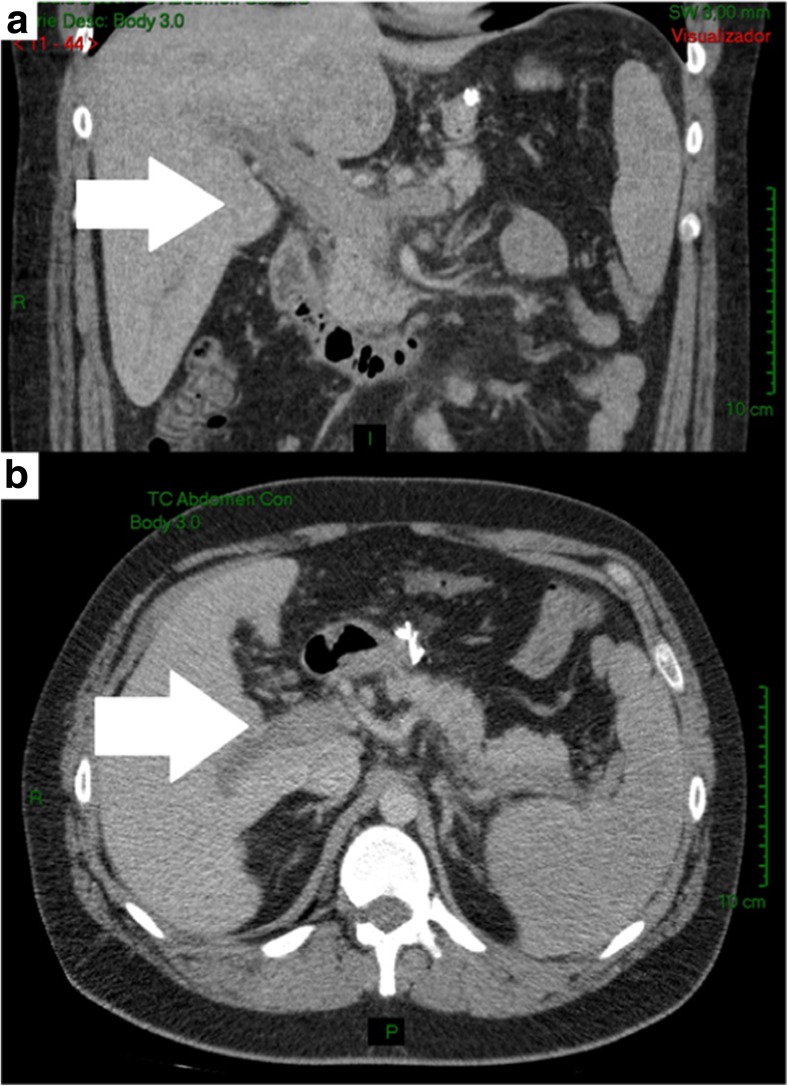
Complete portal thrombosis. CT scan at readmission. **a** Coronal section and **b** axial sections at 12 postoperative day

The partial or complete thrombosis location of the portal vein and its branches were identified (Fig. 1), and all of the patients presented with partial or complete extension to the superior mesenteric vein. Two of the cases even progressed to complete obstruction, although none of them presented with intestinal infarction; those cases showed important development of the collateral vessels. Three patients presented with concomitant thrombosis of the splenic vein. Thrombophilia evaluation was achieved in 3 patients (2 refused to undergo the procedure) 6 weeks after anticoagulant therapy was ended (as suggested by several guidelines). Two patients showed a protein C deficiency.

**Fig. 2:**
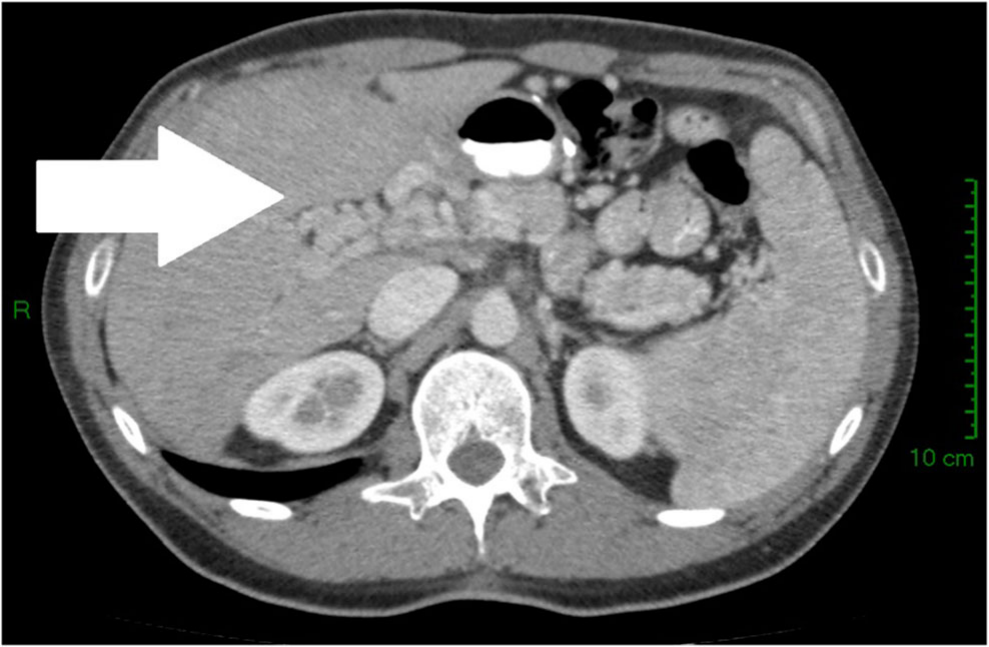
Portal cavernomatosis. CT scan axial section at 20 months after PMVT

After the discharge of patients who developed PMVT, follow-up was conducted using CT and UGE. Two patients showed complete recanalization at the thrombosis site, and three others showed partial recanalization at different degrees. One patient displayed portal cavernomatosis (Fig. 2). No portal hypertension endoscopic findings were observed at a median follow-up of 36 months. The CT images at readmission and follow-up are summarized in Table 4.

**Table 4:** CT scan images at readmission and follow-up

Case	CT POD (days)	iPV^a^				ePV	SMV	pSV	dSV
		LPV	RPV	RaPV	RpPV				
1	15	(–)	T	T	T	Pa75	Pa75	(–)	(–)
	22	(–)	T	T	T	Pa30	Pa30	(–)	(–)
	29	(–)	(–)	(–)	(–)	(–)	(–)	(–)	(–)
	842	(–)	(–)	(–)	(–)	(–)	(–)	(–)	(–)
2	50	Pa30	T	T	T	Pa75	Pa75	(–)	(–)
	93	(–)	T	T	T	Pa30	Pa30	(–)	(–)
	595	(–)	(–)	T	T	(–)	(–)	(–)	(–)
3	11	(–)	T	T	T	Pa50	Pa30	Pa75	T
	15	(–)	T	T	T	Pa50	Pa30	Pa75	T
	21	(–)	T	T	T	Pa50	Pa30	Pa75	T
	747	(–)	(–)	Pa50	T^b^	(–)	(–)	Pa30w/coll	Pa30w/coll
4	12	T	T	T	T	T	T	T	T
	17	T	T	T	T	T	T	T	T
	610	(–)	(–)	(–)	(–)	Cavernomatosis	Tw/coll	Pa80w/coll	Pa80 w/coll
5	16	T	T	T	T	T	T	T	T
	20	T	T	T	T	T	T	T	T
	60	T	T	Pa50	T	Pa30	Tw/coll	Tw/coll	Tw/coll
	89	(–)	(–)	(–)	(–)	(–)	Tw/coll	Tw/coll	Tw/coll

*POD* postoperative day, *T* >90 % thrombosis, *Pa(x)* partial thrombosis percentage of lumen occlusion was calculated by the area of thrombus divided by the area of vein lumen, for more than 90 % was defined as complete, *(*–*)* permeable, *w/coll* with collaterals, *iPV* intrahepatic portal vein, *LPV* left portal vein, *RPV* right portal vein, *RaPV* right anterior portal vein, *RpPV* right posterior portal vein, *ePV* extrahepatic portal vein, *SMV* superior mesenteric vein, *pSV* proximal splenic vein, *dSV* distal splenic vein

^a^CT scan report: segmental portal vein branches according to Couinaud’s classification

^b^Atrophy of the posterior segment VI

## Discussion

The development of venous thrombosis is generally considered to involve a combination of locoregional and systemic prothrombogenic factors [32, 33].

The etiology of PMVT complication after laparoscopic surgery is likely multifactorial. Some of the factors that may contribute to the pathogenesis of this condition include undiagnosed thrombophilia, local injury near the portal flow, surgical damage (including direct trauma leading to diminished blood flow), oral contraceptive use, increased intra-abdominal pressure due to pneumoperitoneum, [14, 16, 20, 26, 34–39] and prolonged time in the reverse Trendelenburg position [18].

PMVT has been well described as a post-splenectomy complication due to hematologic causes, and the symptomatic PVT incidence may vary from 0.2 to 0.9 %, with an increased incidence of up to 10.9 % in asymptomatic patients [19, 40].

In the bariatric field, surgery-related PMVT has been described as an infrequent complication in several case reports and series [11–31]. Multiples hypotheses have been put forward to explain this complication. In this particular group of patients, we included the possible etiologic factors of metabolic syndrome and the thromboembolic risk of obesity itself. In a systematic review, Rocha et al. reported the association between VTE and obese patients undergoing bariatric surgery with an incidence of deep vein thrombosis (DVT) and pulmonary embolism (PE) after surgery varying between 1.2 to 1.6 % and 0.8 to 3.2 %, respectively [39]. Nevertheless, PMVT seems to occur more frequently in patients undergoing LSG than in those undergoing other bariatric procedures.

Specifically, during the LSG procedure, the following contributing factors are considered the most significant:

- Mechanical or thermal effect on the left gastroepiploic arcade or short vessels during the skeletonization of the greater curvature [11]. Intraoperative surgical manipulation may damage the splanchnic endothelium and lead to local thrombus formation that may then propagate throughout the portal venous system [18].
- Blood flow pattern change and diminished flow due to ligation of the short gastric and tributary vessels [18, 20].
- Splenic vein direct contact. Direct contact with the splenic or superior mesenteric vein (SMV) was proposed to be able to occur during surgery and cause thrombosis [11, 26].
- Splenic ischemia or infarction. The ligation of the short vessels during LSG may lead to insufficient perfusion of the upper pole of the spleen, perceptible as a demarcation during the operation. Such a condition, while asymptomatic in most cases, may occasionally lead to the symptomatic development of a splenic infarct and even subsequent abscess, reflecting the release of inflammatory mediators [41–44].
- Dehydration. Liquid intake is limited after bariatric surgery due to the restrictive component of these surgeries, and some patients may be in a negative fluid balance after discharge, which predisposes them to VTE (including PMVT) [11, 20, 26].

The liver retractor used during LSG may cause blood stasis within the liver; the presence of a retrograde thrombosis might be another underlying factor for PMVT [11, 36, 37]. In our series, there was one patient for whom the liver retractor was not used due to anatomic properties such as a small left lobe, and this patient developed PMVT without major liver manipulation during the procedure. Goitien et al., in a multicenter study including 5706 bariatric surgeries, reported 17 (0.3 %) cases of PMVT; all cases but one occurred after LSG, and no PMVT occurred in the group of approximately 1000 patients who had undergone laparoscopic Roux-en-Y gastric bypass (LRYGB) [20]. Additionally, in our complete series of 1578 surgeries, no PMVT was reported outside of the LSG group.

The broad spectrum of clinical presentations of PMVT ranges from incidental findings, in an asymptomatic patient, to life-threatening bowel infarction [13, 14, 17]. Leukocytosis and mild elevation of liver function tests are also observed. Thus, physical examination findings can be normal, or alternatively, if associated with bowel ischemia, patients could present with peritonitis and septic shock [18]. CT enhanced with oral and intravenous contrast has been reported in published studies to diagnose and monitor the patient’s course with a sensitivity of 90 % [13, 14].

Once a diagnosis of PMVT is made, treatment should be promptly started. In patients presenting with peritonitis or shock, an exploratory laparotomy is required with possible resection of necrotic bowel. Full anticoagulation with either subcutaneous LMWH or intravenous unfractionated heparin is applied in patients who do not develop bowel ischemia or necrosis [19, 45]. In patients with recent portal or mesenteric vein thrombosis treated with anticoagulation, not only the follow-up imaging data but also the clinical outcome appears favorable [46]. This treatment is continued and changed to oral anticoagulation (target international normalized ratio, 2.5–3), which should be continued for several months; the duration of this treatment will depend on the coagulation profiles and a hematologic consultation [19, 46].

Early diagnosis and treatment of the underlying causal factors (abdominal sepsis and pro-thrombotic factors) following prompt anticoagulant therapy could lead to a dramatic decrease in the incidence of extrahepatic portal hypertension in the near future [46, 47]. In contrast, late detection and treatment may result in pre-sinusoidal portal hypertension with the associated sequelae and portal cavernomatosis; the latter can result in portal hypertension and complications such as variceal gastrointestinal bleeding [21].

Prophylaxis is routinely perioperatively administered as a standard prevention measure for DVT in most bariatric centers, and many different regimens are used regarding the time of initiation (pre-surgery, anesthesia induction, intraoperatively, and post-surgery), specific pharmacologic agent used, dosage, and length of treatment (during hospital stay until discharge and up to 2 weeks after surgery) [11, 15, 17, 18, 20, 22, 23, 26–31]. However, there is no consensus concerning PMVT prophylaxis.

## Conclusions

PMVT is a rare complication after laparoscopic bariatric surgery, as observed in our series.

A history of smoking was a predominant risk factor for PMVT, and abdominal pain was the main symptom. CT of the abdomen and pelvis is the proven method of choice for PMVT diagnosis. Early diagnosis and appropriate management are essential in preventing catastrophic complications such as intestinal infarction and corresponding increases in morbidity and mortality. Anticoagulant therapy is generally satisfactory to avoid thrombosis progression and to achieve partial or complete recanalization. However, careful follow-up is necessary to evaluate the impact of PMVT on long-term patient outcomes.
